# Persistence of immune response to an adjuvanted varicella-zoster virus subunit vaccine for up to year nine in older adults

**DOI:** 10.1080/21645515.2018.1442162

**Published:** 2018-03-21

**Authors:** Tino F. Schwarz, Stephanie Volpe, Gregory Catteau, Roman Chlibek, Marie Pierre David, Jan Hendrik Richardus, Himal Lal, Lidia Oostvogels, Karlis Pauksens, Stephanie Ravault, Lars Rombo, Gerard Sonder, Jan Smetana, Thomas Heineman, Adriana Bastidas

**Affiliations:** a Laboratory Medicine and Vaccination, Klinikum Würzburg Mitte, Standort Juliusspital, Würzburg, Germany; b Clinical R&D, GSK, Wavre, Belgium; c Biostatistics & Statistical Programming, Clinical Evidence Generation, R&D, GSK, Wavre, Belgium; d Department of Epidemiology, Faculty of Military Health Sciences, University of Defense, Hradec Kralove, Czech Republic; e Biostatistics & Statistical Programming, Clinical Evidence Generation, R&D, GSK, Rixensart, Belgium; f Department of Infectious Disease Control, Municipal Public Health Service Rotterdam-Rijnmond, Rotterdam, The Netherlands; g Clinical R&D, Pfizer Inc., Collegeville, PA, USA; h Medical Sciences, Section of Infectious Diseases, Uppsala University Hospital, Uppsala, Sweden; i Clinical Laboratory Sciences, GSK, Rixensart, Belgium; j Department of Medical Biochemistry and Microbiology, Zoonosis Science Center, Uppsala University, Uppsala, Sweden; k Department of Infectious Diseases, Public Health Service of Amsterdam, Amsterdam, The Netherlands; l Clinical Development, Genocea Biosciences, Cambridge, MA, USA

**Keywords:** herpes zoster (shingles) vaccine, herpes zoster, immunity, persistence, prediction modeling, prevention, subunit gE vaccine, varicella-zoster virus

## Abstract

**Background**: In adults aged ≥60 years, two doses of the herpes zoster subunit vaccine (HZ/su; 50 µg varicella-zoster virus glycoprotein E [gE] and AS01_B_ Adjuvant System) elicited humoral and cell-mediated immune responses persisting for at least six years. We assessed immunogenicity nine years post-initial vaccination.

**Methods**: This open extension study (NCT02735915) followed 70 participants who received two HZ/su doses in the initial trial (NCT00434577). Blood samples to assess the cellular (intracellular cytokine staining) and humoral (ELISA) immunity were taken at year nine post-initial vaccination.

**Results**: Participants' mean age at dose 1 was 72.3 years. The fold increases over pre-vaccination in the mean frequency of gE-specific CD4+ T-cells expressing ≥2 activation markers plateaued from year four post-dose 1 until year nine. Anti-gE antibody geometric mean concentrations plateaued and remained above pre-vaccination levels from year four onwards. Immunogenicity at year nine was similar across age strata (60–69, ≥70 years) and confirmed statistical prediction model results using data for up to year six. Further modeling using all data up to year nine predicted immune responses would remain above the pre-vaccination level up to year 15.

**Conclusion**: In adults aged ≥60 years, HZ/su-induced immunogenicity remained above pre-vaccination levels for at least nine years post-initial vaccination.

**Summary**: After vaccination with HZ/su, both cell mediated and humoral immunity remained above pre-vaccination levels up to year 9 regardless of age group. Immune responses are predicted to remain above baseline up to 15 years post initial vaccination.

**Focus on the Patient**

What is the context?

The reactivation of latent varicella-zoster virus (VZV), leading to herpes zoster (shingles), is more likely to occur in older adults due to aging of the immune system and waning of VZV immunity over time. The immunogenicity of the non-live herpes zoster subunit vaccine (HZ/su) previously showed persistence for at least six years in adults ≥60 years old.

What is new?

HZ/su showed persistent and age-independent VZV-specific humoral and cell-mediated immune responses for nine years in adults ≥60 years old at the time of vaccination, confirming statistical prediction models based on immune responses measured at earlier time points. Both the humoral and cellular median immune responses remained above baseline until the nine year time point in this study.

What is the impact?

The results of this study suggest that HZ/su induces immune responses persisting up to nine years post-initial vaccination. In recent studies, HZ/su showed efficacy for up to 3.7 years. It is not known if these immunological results correlate with long-term protection against HZ. Further results on the persistence of immunogenicity up to 10 years are awaited from the present study, whereas another study is ongoing and will assess the long-term efficacy of the vaccine for up to 10 years.

## Introduction

Herpes zoster (HZ) is a disease caused by reactivation of latent varicella-zoster virus (VZV) that persists asymptomatically in the body after a previous chickenpox episode.^1^ It usually presents as a vesicular rash with a unilateral dermatomal distribution and is almost always accompanied by pain.^2^

Decreased VZV-specific cell-mediated immunity, which may be caused by natural aging-related immunosenescence or immune compromising diseases or treatments, is considered a risk factor for developing HZ.^1^ In immunocompetent individuals, aging is the dominant risk factor for HZ as well as its complications, and the incidence increases substantially from about 50 years of age. The cumulated lifetime incidence of HZ is approximately 30%.^2^

Since 2006, a live attenuated vaccine for prevention of HZ has been licensed for older adults. This vaccine was shown to have a 70% efficacy in preventing HZ in individuals aged 50–59 years,^3^ but the efficacy decreases with age to 37.6% in those aged ≥70 years.^4^ Moreover, in a large observational study including individuals aged ≥60 years, the efficacy of the vaccine decreased from 68.7% in the first year to 4.2% in the eighth year.^5^ Furthermore, as a live attenuated vaccine it is contraindicated for individuals with immunodeficiency and can therefore not be used in individuals considered to be at relatively high risk for HZ .^6,7^

As an alternative to the live attenuated vaccine, a subunit vaccine (HZ/su) containing VZV glycoprotein E (gE) with the AS01_B_ Adjuvant System has been approved in the USA and Canada. gE is a major glycoprotein expressed by VZV^1^ and is essential for viral replication and cell-to-cell spread. It is also a primary target of VZV-specific humoral and cellular immune responses.^8^ The AS01_B_ adjuvant has been shown to induce strong CD4+ T-cell and humoral immune responses to a number of different antigens.^9–11^

In Phase II studies conducted in healthy adults 60 years and older (NCT00434577 and NCT01295320), two doses of HZ/su (50 µg gE combined with AS01_B_) administered two months apart induced strong CD4 T cell and humoral immune responses that persisted substantially above pre-vaccination levels for six years.^12^^,^^13^ Statistical models based on data up to six years (72 months) after the first vaccination predicted that both cellular and humoral immune responses would persist above pre-vaccination levels for up to 10 years.

HZ/su was evaluated in two pivotal Phase III randomized placebo-controlled efficacy trials in individuals aged ≥50 years (ZOE-50; NCT01165177) and ≥70 years (ZOE-70; NCT01165229). These trials showed that HZ/su was highly efficacious in preventing HZ and its complications with an acceptable clinical safety profile.^14^^,^^15^ Notably, the efficacy of HZ/su in preventing HZ did not decrease with increasing age and remained high after a mean follow-up of 3.7 years.^14^^,^^15^

In the present study, we assess the long-term persistence of the cellular and humoral immune responses induced by two vaccination doses nine years (108 months) after the first dose of the initial HZ/su vaccination in the Phase II trial (NCT00434577). These observations were further used to assess the robustness of the predictions of immunogenicity persistence from the above-mentioned statistical prediction models based on follow-up data up to 72 months and to generate additional predictions of immunogenicity persistence up to 15 years after the initial vaccination.

## Patients and methods

### Study design and participants

This is an open-label, long-term follow-up extension study (NCT02735915) of the above-mentioned original Phase II trials (NCT00434577 and NCT01295320). It includes the cohort of subjects in the Phase II trials who received two doses of HZ/su administered two months apart. To assess the nine-year persistence of the immune responses elicited by HZ/su, a blood sample was drawn 108 months after the first dose of the initial vaccination. At this visit, a limited safety follow-up was also performed as part of the recording of the subjects' medical history. The study is ongoing and at month 120, the immunogenicity of HZ/su 10 years after the initial vaccination will be assessed. In addition, the safety and immunogenicity of two booster doses of HZ/su two months apart with the first booster dose administered at month 120 will also be assessed. Here, we report only the results of the assessments at month 108.

Subjects were required to have received two doses of HZ/su two months apart in the initial Phase II trial to be eligible for inclusion in this study. Subjects were excluded, if they: 1) had used any investigational or non-registered drug or vaccine during the 30 days prior to the first study visit; 2) had received or were anticipated to receive immunosuppressant or immune-modifying drugs during the six months prior to study entry or during the entire study period; 3) had any immunosuppressive or immunodeficient condition resulting from disease; 4) had any previous HZ episode.

The study included subjects from three of the four countries participating in the original Phase II trial, Germany, Sweden and the Czech Republic. The study protocol was approved by the ethics committees of each country and was conducted according to the principles originating in the Declaration of Helsinki and following Good Clinical Practice guidelines.

### Assessment of immunogenicity

Cellular and humoral immune responses induced by HZ/su were assessed from blood samples collected at month 108 after the first dose of the initial HZ/su vaccination. We assessed the frequency of CD4 T cells expressing the following activation markers: interferon-γ (IFN-γ), interleukin-2 (IL-2), tumor necrosis factor-α (TNF-α), and CD40 ligand (CD40L). Peripheral blood mononuclear cells (PBMCs) were separated from heparinized blood over Ficoll-hypaque, frozen, cryopreserved and stored at -196°C before testing.^16^ Viability of thawed PBMCs, checked prior to testing by intracellular cytokine staining, was required to be >80%.^17^ PBMCs were stimulated for 2 hours with a pool of 134 15-mer peptides overlapping by 11 (1.25 μg/mL each) spanning the entire gE ectodomain (residues 1 to 546) (Eurogentec), before 18-hour overnight incubation with brefeldin A (1μg/mL) at 37°C. The PBMC preparation was performed by certified operators, and the certification process includes stimulation with SEB to ensure immunocompetence of separated cells. Cells were stained with a viability dye and for phenotypic surface markers (CD4, CD8), fixed, permeabilized, and stained with antibodies to CD3, CD40L, IFN-γ, TNF- α and IL-2. Cells were then washed and analyzed by flow cytometry, as previously described.^17^ CMI responses were expressed as the frequency of viable CD4 T cells expressing two or more activation markers (CD4^2+^ T cells) per 10^6^ CD4 T cells. The cut-off for the analysis was 320 positive cells per 10^6^ CD4 T cells counted. Serum anti-gE antibody concentrations (mIU/mL) were assessed by a GSK in-house enzyme-linked immunosorbent assay with a cut-off value for seropositivity of 97 mIU/mL.

### Statistical analysis

The study was descriptive with no hypothesis tested. The primary objective was to assess the cellular and humoral immune responses 108 months after the first dose of the initial HZ/su vaccination. In a previous study based on immunogenicity data up to month 72 (i.e., blood samples collected at several time points) after the first dose of the initial HZ/su vaccination, three different statistical prediction models^18^ were used to predict the immunogenicity up to 10 years post vaccination.^19^ These predictions are compared descriptively to the immunogenicity observed at month 108.

### Prediction of long-term immune responses by statistical modeling

To extend the prediction of the long-term persistence of the immune responses induced up to 15 years, the individual subject values for all available samples at each time point up to 108 months after the initial vaccination were used to refit three different mixed effects statistical models previously described^18^ and used to predict the immunogenicity up to 10 years after the initial vaccination. The models were fitted separately for the anti-gE antibodies and for the frequencies of CD4 [2+] T cells. The three mixed effects models used were the following.

### Piece-wise linear model

The piece-wise linear model assumes that the immune response declines linearly over time at a rate varying between non-overlapping time intervals. Three break-points were assumed, the two first fixed at 3 and 12 months, respectively, and the last determined based on Akaike's Information Criterion (see^20^) to ensure maximum data fit. For the anti-gE antibodies, the third break-point was 29 months and for the CD4 [2+] T cells it was 49 months. The piece-wise linear model can thus be specified as:

$$\text{f}\left(\text{t}\right)={\text{β}}_{\text{0}}{\text{+ β}}_{\text{1}}\text{t}\;\;\;\;\;\to \;\;\;\;\;\text{if month 3}\leq \text{t}<\text{month 12}$$

$$\text{f}\left(\text{t}\right)\text{=}{\text{β}}_{\text{0}}{\text{+ β}}_{\text{1}}{\text{t + β}}_{\text{2}}\left(\text{t−12}\right)\;\to \text{ if month 12 }\leq \text{ t < month x}$$

$$\text{f}\left(\text{t}\right)\text{=}{\text{β}}_{\text{0}}{\text{+ β}}_{\text{1}}{\text{t + β}}_{\text{2}}\left(\text{t−12}\right){\text{ + β}}_{\text{3}}\left(\text{t −x}\right)\to \text{if t }\geq \text{ month x}$$

where f(t) is log(concentrations/frequencies), t is time (in months) since dose 1 and x is the month to determine (here 29 months for antibody concentrations and 49 months for frequencies). The β_i_ (i = 1,2,3) are the parameters corresponding to the respective time intervals.

### The power-law model

The power-law model includes the rate of B cell decay to model the biological dynamics of the immune response assuming a logarithmic function to estimate the persistence over time of anti-gE antibody levels or frequencies of CD4 [2+] T cells. The power-law model is written as:

f(t) = k−a log (c + t)

where k is the peak log level, a is the decay rate over time, and c is an arbitrary small constant (often set to 0).

### The modified power-law (Fraser) model

The Fraser model^21^ is a modification of the power-law model, which takes into account two populations of B cells, namely activated and memory B cells that are involved in a long-term plateau of the immune response. The Fraser model is written as:

$$\text{f}\left(\text{t}\right)\text{ = k + log }(\left(\text{1−π}\right){\text{t}}^{\text{−}}{}^{\text{a}}\text{+ π})$$

where π (between 0 and 1) indicates the relative level of immune response produced in the long-term memory plateau. A value of π different from 0 indicates long-term persistence of the immune response. The model assumes that the amount of antibodies produced by the activated B cells decreases over time, whereas that produced by the memory B cells is constant over time.^21^

Regardless of the prediction model used, log antibodies/frequencies are modeled providing predicted geometric mean concentrations (GMCs) of antibodies and geometric means (GMs) for the frequency of CD4[2+] T cells. The statistical analyses were performed using SAS®.

## Results

### Subjects

The original Phase II trial was carried out in Germany, Sweden, the Czech Republic and the Netherlands. Of the 714 subjects vaccinated, 166 were assigned to the HZ/su cohort and 147 completed the study to month 36.^12^ Of these, 129 were enrolled and 119 completed the follow-up study to 72 months.^13^ Subjects from centers in the Netherlands were not offered participation in the present study, since only three of the 14 Dutch subjects participating in the follow-up study at 72 months^13^ would have been eligible in the present study; for logistical reasons it was not considered feasible to include them.

Seventy subjects in two age cohorts (60 to 69 and ≥70 years at the time of the initial vaccination) met the eligibility criteria and consented to participate. The demographic characteristics of this cohort were similar to those of the cohort participating in the 72 months follow-up study and to those of the cohort enrolled in the initial trial. Their mean age at first dose of the initial vaccination was 72.3 years, 57 (81%) were ≥70 years old, 43 (61%) were women and all were Caucasians (Table 1).

**Table 1.** Demographic characteristics of the participants in the study at the time of the initial vaccination.

Characteristic	Category/parameter	HZ/su group (n = 70)
Age at first dose of initial vaccination (years)	Mean (SD)Range	72.3 (4.3)61–81
Age category, n (%)	60–69 years≥70 years	13 (18.6)57 (81.4)
Sex, n (%)	FemaleMale	43 (61.4)27 (38.6)
Ethnicity, n (%)	White-Caucasian	70 (100)

### Cell-mediated immune response

**Figure 1.**
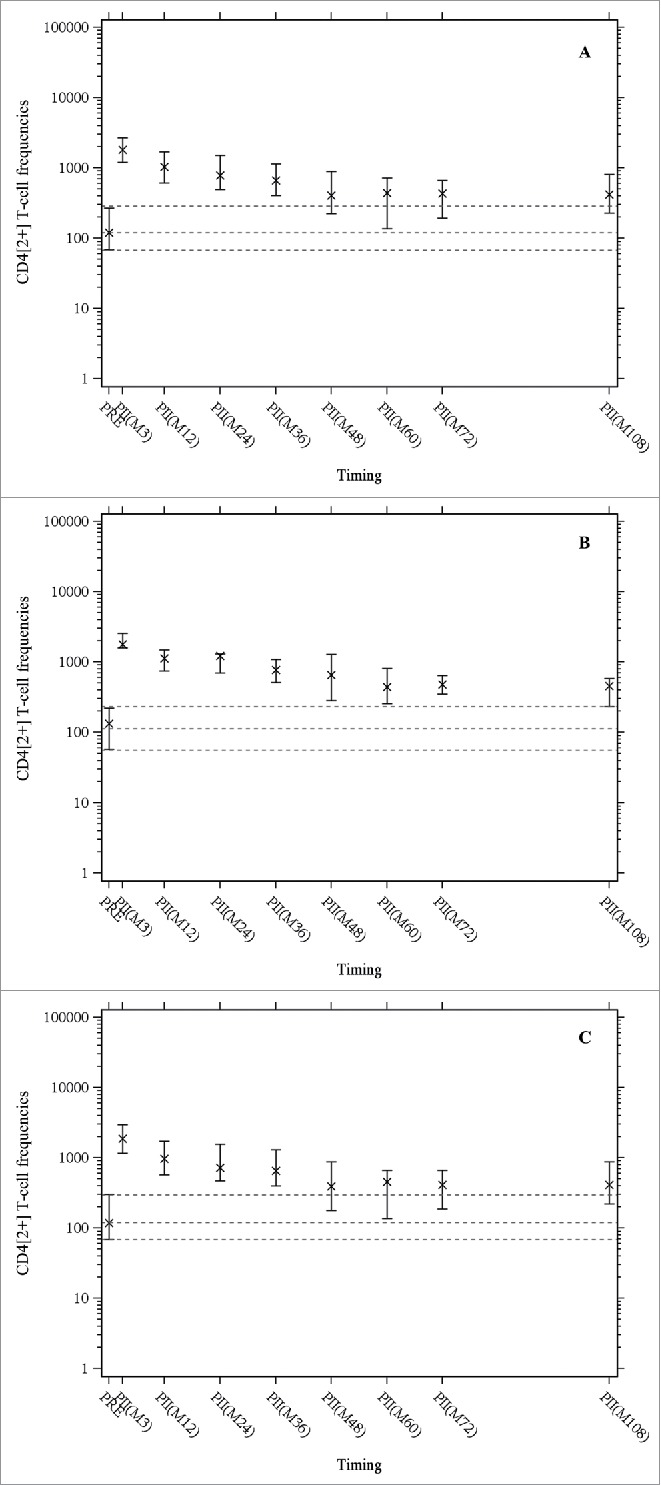
The median frequencies of CD4+ T cells expressing at least two activation markers (among CD40 ligand, interleukin-2, tumor necrosis factor-α, and interferon-γ) per 10^6^ cells after in-vitro stimulation with gE were measured by intracellular cytokine staining followed by flow cytometry. X indicates medians, the horizontal bars indicate Q1 and Q3, dashed lines indicate pre-vaccination values (Q1, median, Q3); PRE = before vaccination; PII(Mxx): xx months after the first dose of vaccination. Panel A: Overall population; Panel B: age cohort 60 – 69 years; Panel C: age cohort ≥ 70 years.

At month 108, the median gE-specific CD4[2+] T cell frequency was 415.4 [Q1-Q3: 227.1–803.9], similar to the frequency at month 72 (429.7, Q1-Q3: 192.5–659.0) and higher than the pre-vaccination frequency (120.6, Q1-Q3: 68.9–269.6) (Fig. 1A). The median gE-specific CD4[2+] response was 3.4 times higher than the pre-vaccination value, the same fold-increase as at month 72. In both age cohorts, the median frequencies of gE-specific CD4[2+] T cells were the same at month 108 as at month 72 (Fig. 1B/C).

### Humoral immune responses

**Figure 2.**
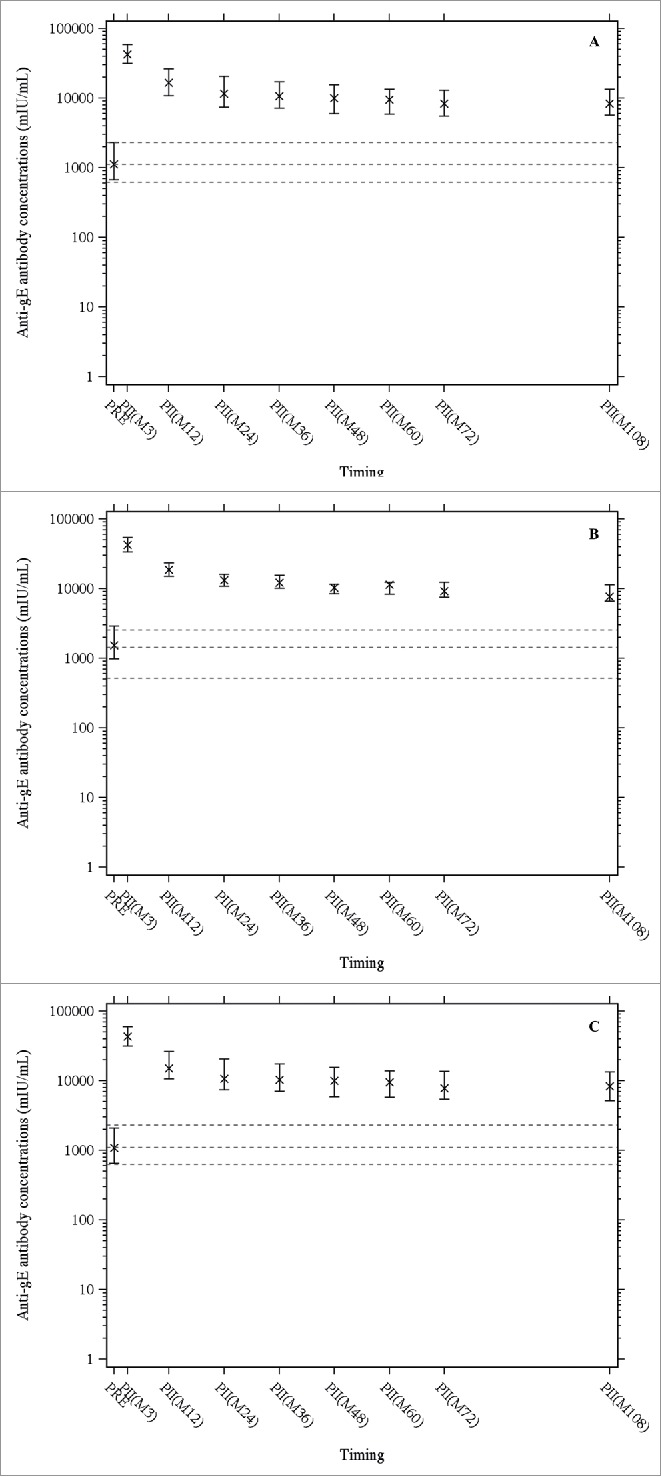
Anti-gE antibody concentrations were determined by enzyme-linked immunosorbent assay. X indicates medians, the horizontal bars indicate Q1 and Q3, dashed lines indicate pre-vaccination values (Q1, median, Q3); PRE = before vaccination; PII(Mxx): xx months after the first dose of vaccination. Panel A: Overall population; Panel B: age cohort 60 – 69 years; Panel C: age cohort ≥ 70 years.

All subjects were seropositive for anti-gE antibodies prior to vaccination and remained seropositive at all time points. At month 108, the median anti-gE antibody concentration was 8345 mIU/mL (Q1-Q3: 5709–13513), similar to the value at month 72 (8362, Q1-Q3: 5549–12985) (Fig. 2A). The median anti-gE antibody concentration at month 108 was 7.4 times higher than the pre-vaccination concentration (1121, Q1-Q3: 671–2309), the same fold increase as at month 72. (Fig. 2 B/C).

### Safety

At the month 108 study visit, no vaccine-related serious adverse events or suspected HZ episodes were reported during the medical history recording.

### Comparison with the predictions of the statistical prediction models

All three statistical prediction models (piece-wise linear, power-law and Fraser) based on observed data up to 72 months after the initial vaccination predicted that both cell-mediated and humoral immune response would persist (remain above the pre-vaccination levels) up to at least 10 years after the vaccination. The observed immune response data at month 108 confirm these model predictions up to nine years after the vaccination and with no indication of any substantial decline since the assessment at four years (Figs. 1 and 2).

### Prediction of immunogenicity up to 15 years

**Figure 3.**
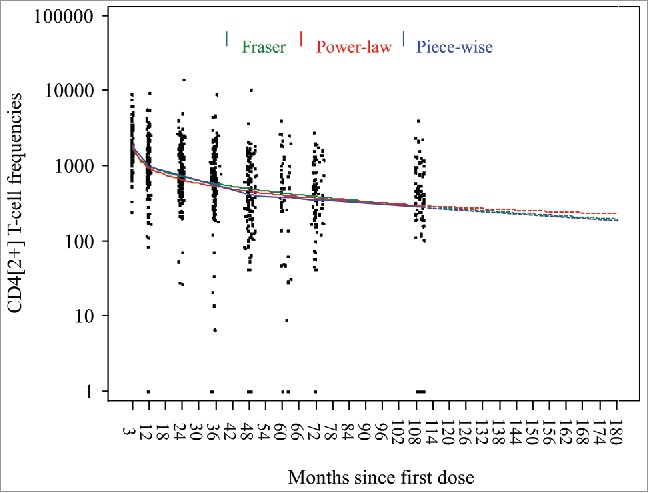
Predicted geometric means of frequencies of gE-specific CD4[2+] T cells based on three statistical prediction models (piece-wise linear, power-law, Fraser).

**Figure 4.**
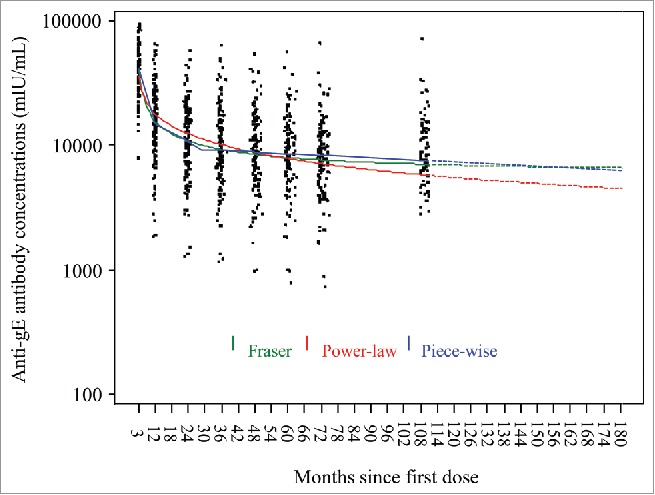
Predictions of anti-gE antibody geometric mean concentrations based on three statistical prediction models (piece-wise linear, power-law, Fraser).

Further modeling based on adding the observations at month 108 indicates that for gE-specific CD4[2+] T cells, predicted GM frequencies will remain above the GM frequency observed pre-vaccination at least 15 years after the initial vaccination (Fig. 3). For anti-gE antibodies, predicted GMCs also will remain above the GMCs observed pre-vaccination at least 15 years after the first dose of vaccination (Fig. 4).

### Prediction model results up to year 10 based on month 108 data versus month 72 data

**Figure 5.**
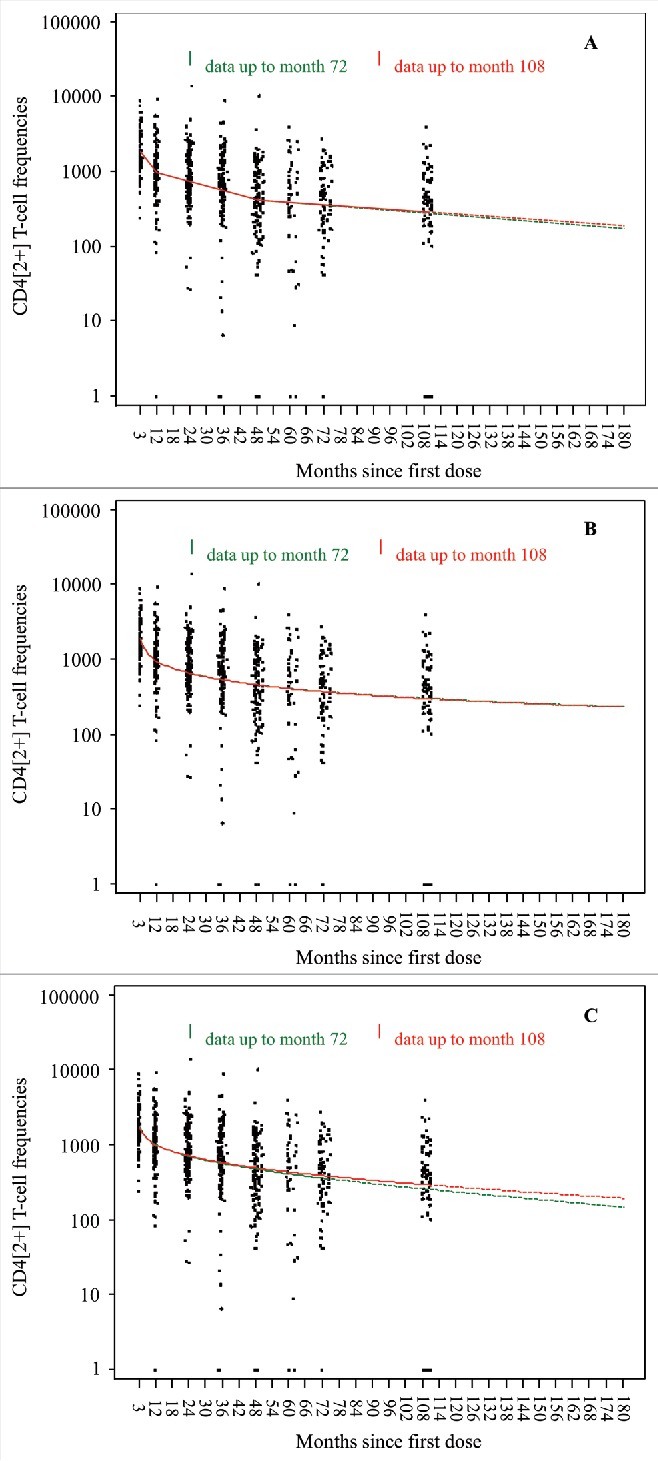
Prediction models for frequency of gE-specific CD4[2+] T cells 72 months vs 108 months (Subjects who received two doses of HZ/su VZV vaccine). Panel A: Piece-wise linear; Panel B: Power-law, Panel C: Fraser.

**Figure 6.**
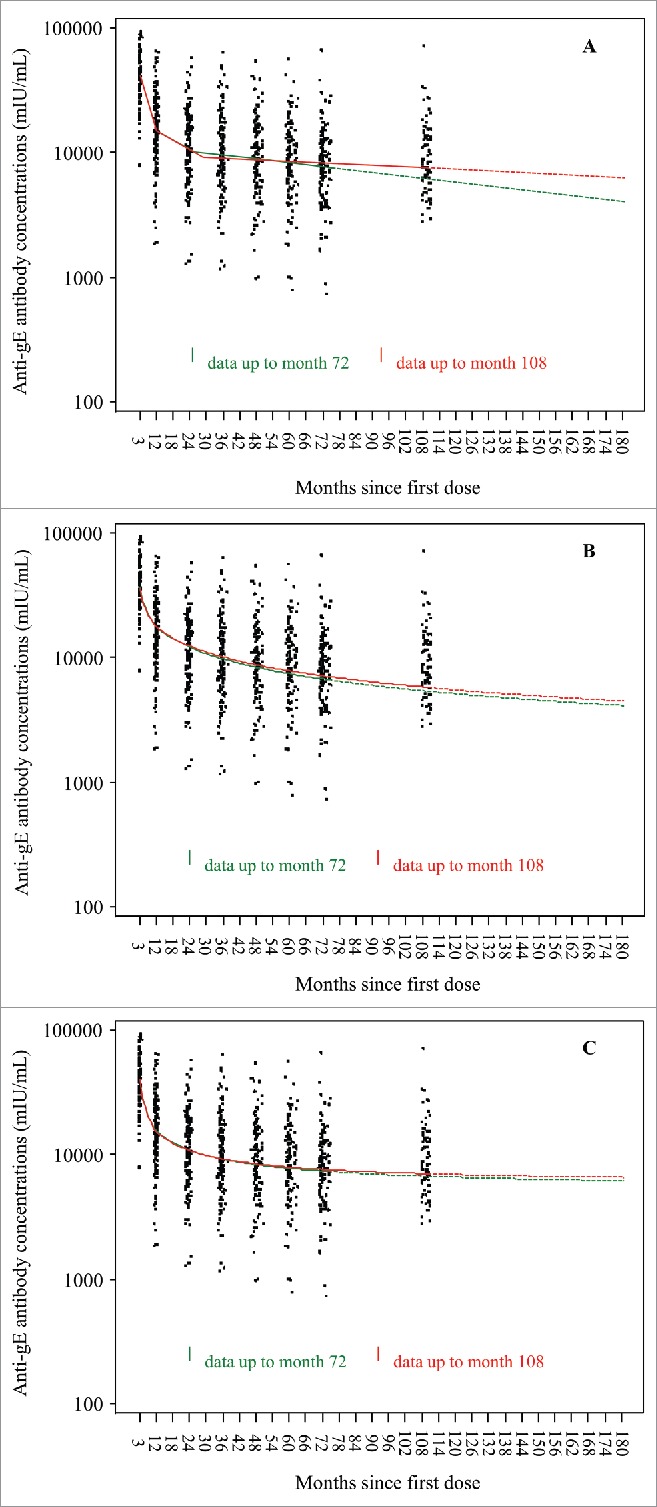
Prediction models for anti-gE antibodies 72 months vs 108 months (Subjects who received two doses of HZ/su VZV vaccine). Panel A: Piece-wise linear; Panel B: Power-law; Panel C: Fraser.

For the prediction up to year 10 of gE-specific CD4[2+] T cells, the curves derived by including the observed data at month 108 to the models are similar to those based on observed data until month 72 for the piece-wise linear and the power-law models and slightly higher after month 72 for the Fraser model (Fig. 5). For the anti-gE antibodies, the curves derived with the follow-up to month 108 are similar to those based on data until month 72 for the power-law and Fraser models whereas the month 108 follow-up data leads to moderately higher predicted GMCs with the piece-wise linear model (Fig. 6).

## Discussion

This study provides data on the persistence of immune responses to HZ/su up to 108 months after initial vaccination of older adults, adding to the previously reported 36 and 72 months results.^12^^,^^13^ Nine years after the initial vaccination, both cell-mediated and humoral immune responses to HZ/su remained at approximately the same level as at six years after vaccination. It is observed that both immune responses plateau as of around four years post-vaccination. At year nine, the CD4[2+] response remained at least three-fold above the pre-vaccination level and the humoral response seven-fold above, and both immune responses were similar in the two age cohorts, 60–69 and ≥70 years. No vaccine-related SAEs or suspected HZ breakthrough episodes were reported during the medical history recording, when the blood samples were drawn at month 108.

The immunogenicity results observed at nine years after the initial vaccination validated the results of mixed effects statistical prediction models based on immunogenicity data up to six years post-vaccination. The immunogenicity data obtained in the present study were added to the same prediction models to generate predictions up to 15 years post-vaccination, and these show that both humoral and cellular immune responses are predicted to remain above pre-vaccination levels at 15 years. The immunogenicity data to be collected at year 10 may be used to validate these extended predictions.

To date, an immunological threshold of protection for HZ has not been established.^22^ Therefore, the level of immune responses observed in this study does not imply that vaccinated subjects will be clinically protected against HZ for nine years after vaccination. Additional follow-up and booster doses are planned at year 10 to gather further information on the waning profile of HZ/su and determine whether a booster dose will be needed. At baseline, based on the presence of anti-gE antibodies, all the 70 subjects had serological evidence of past exposure to VZV indicating that they were susceptible to develop HZ but no case of confirmed or suspected HZ was reported over the nine year period. Furthermore, the cell-mediated immune responses of the vaccinees remained at the same level from month 48 to month 108 post-vaccination, whereas the increase in age for non-vaccinated people over 60 would be expected to be accompanied by a decrease in cell-mediated immune responses.^1,23^ Subclinical reactivation of VZV is one of the mechanisms some experts believe contributes to maintain a certain level of immune response, which then prevents clinical reactivation. These subclinical reactivations, as a natural source of periodic boosting, can happen throughout a patient's lifespan which would include the time period before the subjects entered the study.^24^ Therefore, since our year 9 data shows that the immune level is higher than the baseline level, it is likely the effect of the vaccination rather than subclinical reactivation of VZV.

The study was not without limitations. The sample size was small resulting in less precise estimates of the immune responses and limited probability of detecting rarer adverse effects of vaccination. Although it is not expected that new adverse effects of the vaccine would be observed between six and nine years post-vaccination.

The original Phase II trial excluded subjects with any immunocompromising conditions and also any clinically significant acute or chronic pulmonary, cardiovascular, hepatic or renal functional abnormality. Eligibility for the present study was conditional on absence of any immunocompromising condition or therapy, so the study cohort consisted of older adults who may not be fully representative of the target population of all older adults.

Furthermore, the study included only participants who had received two doses of the selected formulation of HZ/su, so there was no control group. The average immune response was assessed by comparison with the average pre-vaccination levels of cellular and humoral immunity. The study was conducted in three northern European countries and all the participants were Caucasian. Populations in other geographic areas or of other ethnicities not included in this study may potentially respond differently to the HZ/su vaccine. This was however not observed in the two pivotal Phase III studies as based on a well-defined and globally consistent disease confirmation method. The ZOE-50 and ZOE-70 trials showed that the efficacy of HZ/su in preventing HZ in adults aged ≥50 years was consistently very high across all study regions.^25^

In conclusion, the immune responses observed nine years after the first dose of the initial vaccination with HZ/su were similar to those observed after six years. Median cell-mediated immune responses remained more than three-fold above the pre-vaccination level and median humoral immune responses seven-fold, both immune responses were stable from month 72 to month 108, and were maintained regardless of age group. These observations confirmed the predictions of statistical models fit to the observed immunity data up to six years after the vaccination. The three prediction models used were based on different assumptions but all led to similar predictions, which have now been confirmed with immunological data. Furthermore, the model predictions were not substantially altered by adding data from a new time point three years after the previous one. Based on all the observed data now available, the models predict that the immune response will persist for at least 15 years following the vaccination.
